# Synergistic Interactions between Selected β-Lactam Antibiotics and Cinnamic Acid and Its Chosen Derivatives

**DOI:** 10.3390/antibiotics13080710

**Published:** 2024-07-29

**Authors:** Tomasz Zawiła, Denis Swolana, Marta Zawiła, Robert D. Wojtyczka

**Affiliations:** Department of Microbiology and Virology, Faculty of Pharmaceutical Sciences in Sosnowiec, Medical University of Silesia, ul. Jagiellońska 4, 41-200 Sosnowiec, Poland; d200969@365.sum.edu.pl (T.Z.); dswolana@sum.edu.pl (D.S.); mmkwasniewska@gmail.com (M.Z.)

**Keywords:** antimicrobial activity, biofilm, cinnamic acid, *Staphylococcus epidermidis*

## Abstract

*Staphylococcus epidermidis*, a component of human microbiota, may also cause life-threatening opportunistic infections. These are becoming increasingly common infections associated with the implantation of various implants. Due to the exhaustion of antibiotic resources, new substances with antimicrobial activity are being sought. The present study examined the antibacterial effect of cinnamic acid and its derivatives and their combinations with β-lactam antibiotics on the growth of *Staphylococcus epidermidis* strains isolated from vascular infections. The data obtained during the research indicated that cinnamic acid and its derivatives, sinapic acid, ferulic acid, and p-coumaric acid, have weak antibacterial activity (MIC values at the level of 2048 and 4096 mg/L). The combination of cinnamic acid and its derivatives with β-lactam antibiotics increases the effectiveness of their action and may demonstrate various pharmacological effects depending on the established cutoff.

## 1. Introduction

*Staphylococcus epidermidis* (*S. epidermidis*) is a component of human skin microbiota. It hinders the adhesion of potential pathogens, ensures skin homeostasis, and takes part in promoting immune reactions. It stimulates human keratinocytes to secrete antimicrobial peptides during *Staphylococcus aureus* (*S. aureus*) infection. These peptides, in the presence of *S. epidermidis*, also act synergistically against *Streptococcus pyogenes (S. pyogenes)*. The presence of *S. epidermidis* may also have a positive effect on skin regeneration [1,2]. This microorganism has been “underestimated” for years and treated as a contaminant or commensal flora, but now it is becoming an increasingly important pathogen associated with hospital infections.

The cysteine protease EcpA, present genotypically in all *S. epidermidis* strains, is a recently discovered enzyme showing sequence and structural similarity to Staphopain A and B found in *S. aureus*. This enzyme is responsible for penetrating the skin barrier and stimulating the inflammatory process [1]. In addition to proteolytic enzymes, *S. epidermidis* also contains pathogenicity islands in its genome and has a genotypic ability to produce at least two enterotoxins [3].

*S. epidermidis* is also known for its ability to adhere and form biofilms. It is a “bacterial community” composed of bacteria of one (or many) species, suspended in an extracellular polysaccharide matrix (EPS). It is most often found on medical equipment, such as vascular catheters, dialysis catheters, valve and joint implants, and heart pacemakers. The structure of the biofilm becomes unstable during growth and leads to the dissemination of bacteria to tissues and organs, causing a systemic infection. The poor prognosis for patients (especially non-immunocompetent) is additionally aggravated by the high or very high percentage of multidrug-resistant strains [4]. According to WHO forecasts, in 2050, up to 10 million people will die annually due to infections caused by multidrug-resistant microorganisms. Therefore, it seems appropriate to search for new antimicrobial drugs and combinations of these with other substances in order to counteract the growing drug resistance.

**Figure 1:**
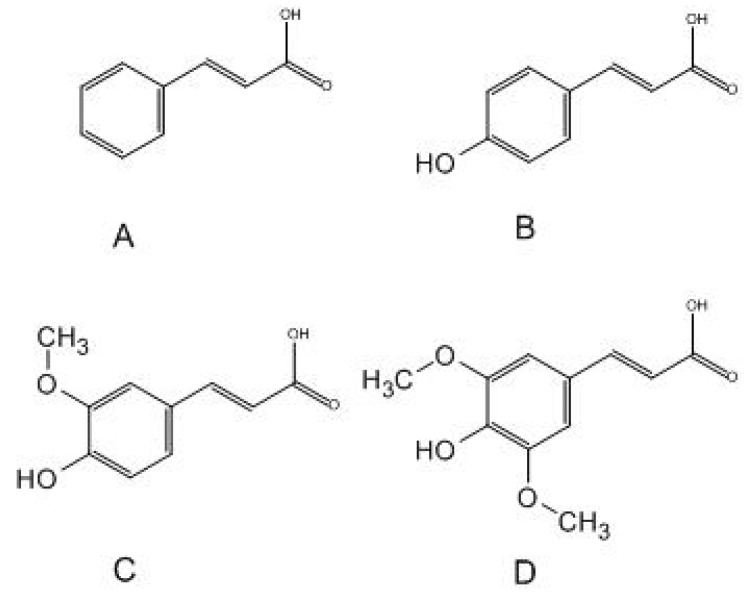
Structural formulae of selected acids: (**A**)—cinnamic acid; (**B**)—p-coumaric acid; (**C**)—ferulic acid; (**D**)—sinapic acid.

Cinnamic acid and its derivatives (ferulic acid, coumaric acid, and sinapic acid) are compounds widely distributed in the plant world. They are important in their physiology and are also metabolites in the biosynthesis of such compounds as anthocyanins, coumarins, flavonoids, stilbenes, and tannins. These play a significant role in the growth and reproduction of plants and have defensive functions [5]. They also have several effects: antibacterial [6], anti-inflammatory, antioxidant [7], neuroprotective, and antidiabetic [8]. They are used in cosmetology as a component of UV filters [5]. In terms of chemical structure, they are simple monocarboxylic acids connected to an acrylic chain. The simplest of them is cinnamic acid. The introduction of a hydroxyl group into the molecule results in the formation of coumaric acid, while the introduction of hydroxyl and methoxy groups allows for the formation of ferulic acid. Sinapic acid is distinguished from ferulic acid by an additional methoxy group at the C5 carbon. The chemical structure of the compounds is shown in Figure 1. Cinnamic acid derivatives are used in cosmetics as perfuming, masking, flavoring, antioxidant, hair care toning, and antimicrobial ingredients [5].

The broad antibacterial activity of these compounds is one of the aspects often discussed by scientists. Their spectrum of activity includes Gram-positive cocci, Gram-negative *Enterobacterales*, non-fermentative bacilli, yeast-like fungi, and molds [6,9,10]. There are also studies indicating their antituberculosis [11] and antiparasitic activity [12,13,14].

The aim of this study is to investigate the impact of antibiotics, cinnamic acid and its derivatives, and their combinations on bacterial populations. Our hypothesis posits that the combination of these substances may induce synergistic effects, enhancing antimicrobial efficacy. We also aim to assess the influence of cutoff values on the interpretation of the obtained data.

## 2. Results and Discussion

### 2.1. Minimum Inhibitory Concentration (MIC) of Selected Antibiotics

**Figure 2:**
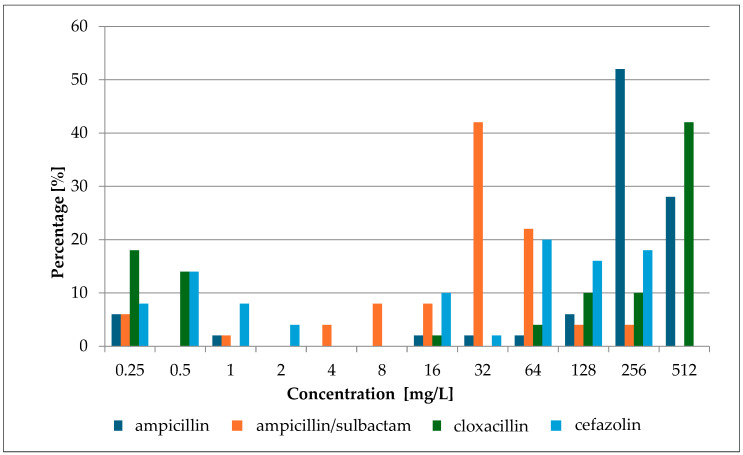
MIC percentage composition for ampicillin, ampicillin/sulbactam, cloxacillin, and cefazolin.

In this study, β-lactam antibiotics with activity against Gram-positive cocci were chosen for experiments. According to the recommendations of the European Committee on Antimicrobial Susceptibility Testing (EUCAST), the therapeutic value of β-lactams is determined by a screening test with a cefoxitin disc (30 µg) [15]. For the purposes of further work, it was necessary to determine the MIC values of selected antibiotics for all strains. The obtained values are shown in Figure 2 and Table S3.

The conducted studies showed a wide range of growth-inhibiting concentration values. The lowest average value for all tested strains was obtained for ampicillin with sulbactam (47.85 ± 51.940 mg/L; 48.20 ± 52.300 mmol/L) and the highest for cloxacillin (376.94 ± 181.925 mg/L; 1.00 ± 0.483 mmol/L). High values were also obtained for the first-generation cephalosporins, cefazolin (104.82 ± 93.986; 0.23 ± 0.207 mmol/L), and for ampicillin (304.7 ± 152.147 mg/L; 0.82 ± 0.409 mmol/L). Cloxacillin and cefazolin play an important role in the treatment of staphylococcal infections or perioperative prophylaxis. Cloxacillin is the drug of choice for infections of the skin, soft tissues, and bones (etiologically caused by *Staphylococcus* spp.), while cefazolin is administered as part of perioperative prophylaxis [16,17].

The issue of resistance of *S. epidermidis* strains to aminopenicillins was analyzed by the team of Siriwong et al. [18]. Four strains of *S. epidermidis*, belonging to the DMST collection, were analyzed. All strains were resistant to both penicillin and amoxicillin, which is structurally similar to ampicillin. MIC values of 200 mg/L (0.599 mmol/L) for penicillin and 16 mg/L (0.044 mmol/L) for amoxicillin were obtained. It should be emphasized that these studies were carried out on a small group of reference strains, not on clinical strains.

The work carried out by Ferraz et al. used ampicillin as a reference point for further analyses [19]. Following The Clinical & Laboratory Standards Institute (CLSI) guidelines, an ampicillin MIC of 0.05 mM (~17.5 mg/L) for *S. epidermidis* (clinical isolate) and 0.005 mM (~1.75 mg/L) L) for *S. aureus* (ATCC 25923) were obtained. The MIC values were relatively high and correlated with the values obtained in this study.

The obtained results confirm the research conducted by Xiaoliang Ba et al. [20] for over 200 strains. The analysis of the activity of aminopenicillin-inhibitor combinations against *S. epidermidis* showed a high percentage of resistant strains and comparable MIC values.

Epidemiological studies carried out in a hospital environment showed a high percentage, approximately 50%, of strains resistant to the combination of ampicillin and sulbactam against coagulase-negative staphylococci [21].

A comparison of the drug resistance profile of *S. epidermidis* strains in two different orthopedic centers was carried out by Stevoska et al. [22]. *S. epidermidis* accounted for 70.5% of the 132 isolates. In the first examined center, 94.7% of isolates were resistant to ampicillin, while in the second one, 89.5% of strains were resistant to this antibiotic. The difference between both centers was not statistically significant (*p* = 0.264). According to the thesis put forward by Stevoska et al., the sensitivity of strains to cefazolin can be estimated on the basis of their sensitivity to oxacillin. It was determined that 62.7% of strains in the first center and 40.4% of isolates in the second center were susceptible to oxacillin. This difference was statistically significant (*p* = 0.011). The data obtained by researchers regarding the antibiotic resistance profile are consistent with the results obtained in this study.

Other studies, conducted by Mirzaei et al., analyzed MIC concentrations for both cefazolin and cloxacillin [23]. This analysis was performed on a large group of strains (159) obtained from hospitalized patients. Cloxacillin MICs ranged from 0.5 to 512 mg/L. Growth inhibitory concentrations obtained for cefazolin were found to range from 0.125 to 256 mg/L. The MIC values are consistent with the results obtained in our study.

### 2.2. Minimum Inhibitory Concentration (MIC) of Cinnamic Acid and Derivatives

**Figure 3:**
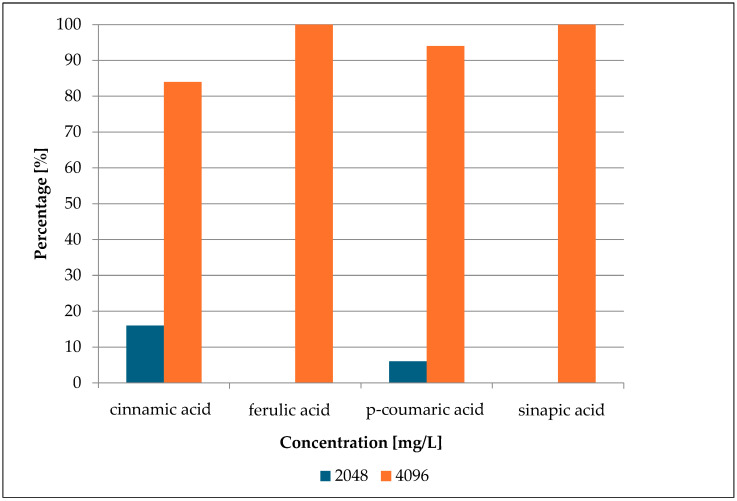
MIC percentage composition for cinnamic acid, ferulic acid, p-coumaric acid, and sinapic acid.

Concurrently, sensitivity testing was conducted on fifty selected strains of *S. epidermidis* against cinnamic acid and its derivatives. These compounds undoubtedly exhibited antimicrobial activity. However, it is worth noting that high concentrations were required to achieve an inhibitory effect >90%. The obtained MIC values of cinnamic acid and its derivatives are presented in Figure 3 below and Table S2.

The research findings revealed that in 84% of cases, cinnamic acid exhibited a growth inhibitory effect only at a high concentration—4096 mg/L (27.7 mmol/L). p-Coumaric acid showed growth inhibition of microorganisms at such a concentration (4096 mg/L; 29.9 mmol/L) at 96%. Lower inhibitory concentrations were observed at 16% for cinnamic acid (2048 mg/L; 13.8 mmol/L) and 4% for p-coumaric acid (2048 mg/L; 10.6 mmol/L). Ferulic acid and sinapic acid exhibited growth inhibition in 100% of cases at the concentration of 4096 mg/L. The inhibitory molar concentration of ferulic acid was 21.0 mmol/L, while that of sinapic acid was 18.7 mmol/L.

The high MIC values of cinnamic acid and its selected derivatives are confirmed by many researchers. Mandal et al. [24] determined the antimicrobial activity of cinnamic acid, p-coumaric acid, and ferulic acid. The studied strain was the reference isolate *S. epidermidis* NCIM 2493. They obtained an MIC value of 512 mg/L for all three acids. It is worth noting that the isolate was a reference strain, not associated with the hospital environment. In the study conducted by Auezova et al. [25], no effect of ferulic acid or p-coumaric acid on *S. epidermidis* cultures was observed. However, the researchers applied only low concentrations of both acids. The conducted work confirmed their relatively weak antibacterial properties.

Research by Taofiq et al. [26] indicated that for various species of Gram-positive bacteria, the MIC values are around 1000 mg/L for cinnamic acid and from 500 to 1000 mg/L for coumaric acid. Antibacterial activity studies were conducted on strains of *S. aureus*—MRSA (methicillin-resistant *Staphylococcus aureus*) and MSSA (methicillin-susceptible *Staphylococcus aureus*)—as well as *Enterococcus faecalis* strains. Although the study group included only three strains, all were isolated from the hospital environment. These studies confirmed the low antimicrobial activity of the tested acids and the results obtained in this work.

The determinations made by Letsididi et al. indicated a high MIC value of cinnamic acid equal to 6.250 mg/L (0.042 mmol/L) for *S. aureus* strains [27]. In his research, the author mentioned the synergistic effect of the combination of cinnamic acid and antibiotics on the inhibition of mycobacterium bacteria growth.

The MIC value of ferulic acid was also determined as part of the research conducted by Petrișor et al., where the MIC value was determined to be 10,000 mg/L (51.4 mmol/L) for the *S. aureus* ATCC 25923 strain [28]. This is a very high value, difficult to achieve in solution, confirming the weak activity of this acid. The antibacterial and antibiofilm use of hydrogels in therapy was investigated by Xiaoliang et al. [29] and Yajing et al. [30]. The hybrid hydrogel showed remarkable effectiveness in inhibiting *Pseudomonas aeruginosa* biofilm, achieving an inhibition rate of 98% [29]. In turn, the SCE2 hydrogel showed superior photothermal antibacterial capability against *S. aureus* and *P. aeruginosa* [30].

Borges et al. presented different results. Evaluating the activity of ferulic acid against the *S. aureus* CETC 976 strain, they demonstrated an MIC value of 1.1 mg/L (0.0057 mmol/L) [31]. In subsequent studies by Engels et al., an MIC value of 300 mg/L (1.34 mmol/L) was determined for sinapic acid against the *S. aureus* strain [32].

### 2.3. Fractional Inhibitory Concentration Index (FICi) of Selected Antibiotics and Cinnamic Acid and Derivatives

To evaluate the pharmacological effects of combining two substances (selected β-lactam antibiotics and cinnamic acid and its selected derivatives), the Fractional Inhibitory Concentration Index (FICi) value must be determined. This value is typically interpreted for inhibiting microbial growth by 50% or 90% of the control growth value. In this study, the FICi value was assessed for 50%, 75%, and 90% inhibition of microbial growth. This allowed for a comprehensive assessment of pharmacological effects and revealed the influence of the cut-off value on result interpretation [33].

**Figure 4:**
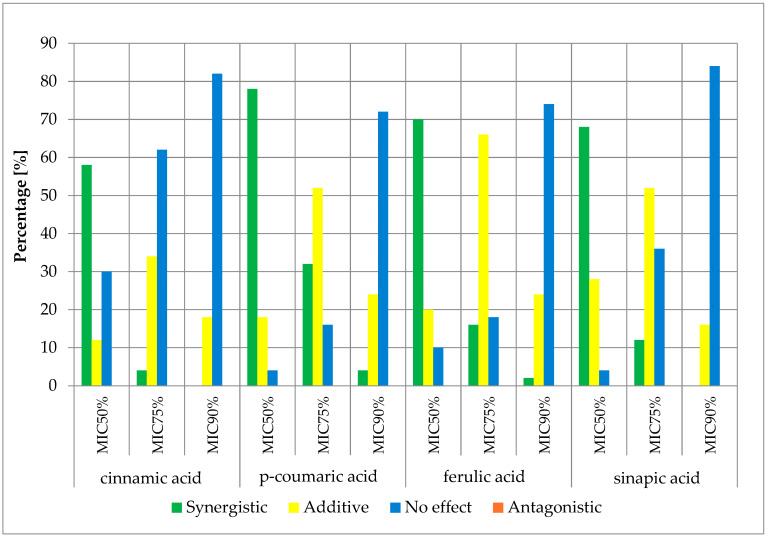
FICi percentage of strains showing a given pharmacological effect for cinnamic acid, ferulic acid, p-coumaric acid, and sinapic acid in combination with ampicillin for three different cut-off values (MIC50%—50% growth inhibition; MIC75%—75% growth inhibition; MIC90%—90% growth inhibition).

**Figure 5:**
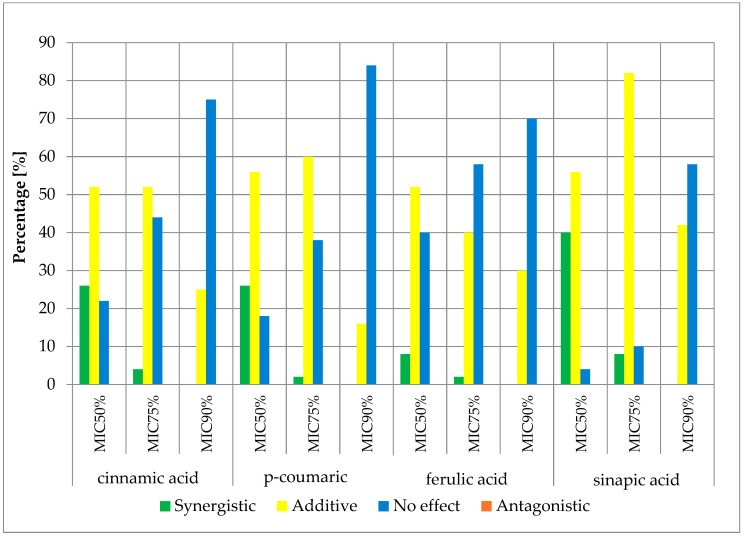
FICi percentage of strains showing a given pharmacological effect for cinnamic acid, ferulic acid, p-coumaric acid, and sinapic acid in combination with ampicillin/sulbactam for three different cut-off values (MIC50%—50% growth inhibition; MIC75%—75% growth inhibition; MIC90%—90% growth inhibition).

**Figure 6:**
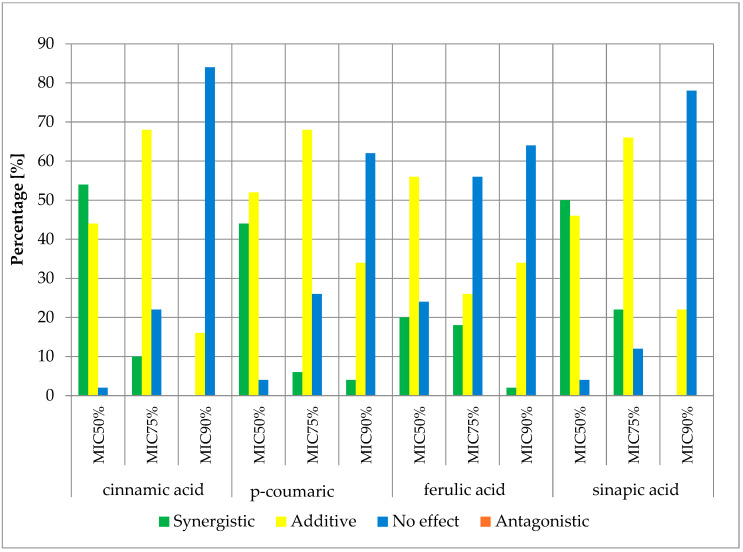
FICi percentage of strains showing a given pharmacological effect for cinnamic acid, ferulic acid, p-coumaric acid, and sinapic acid in combination with cefazolin for three different cut-off values (MIC50%—50% growth inhibition; MIC75%—75% growth inhibition; MIC90%—90% growth inhibition).

**Figure 7:**
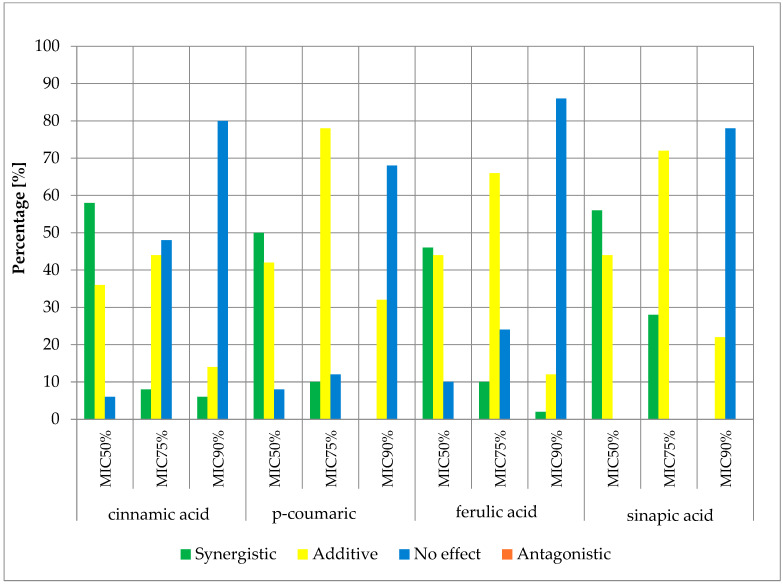
FICi percentage of strains showing a given pharmacological effect for cinnamic acid, ferulic acid, p-coumaric acid, and sinapic acid in combination with cloxacillin for three different cut-off values (MIC50%—50% growth inhibition; MIC75%—75% growth inhibition; MIC90%—90% growth inhibition).

The obtained results of the effects of cinnamic acid and its derivatives in combination with β-lactam antibiotics are presented in the figures below (Figure 4, Figure 5, Figure 6 and Figure 7) and Table S1.

The obtained cumulative results of all determinations for all combinations of antibiotics and cinnamic acid derivatives illustrate the crucial influence of the cut-off value on the obtained outcomes. At the MIC50 cut-off level, a synergistic effect was achieved in 47.00% of cases, an additive effect in 41.13% of cases, and no effect was observed in 11.88% of cases. At the MIC75 cut-off level, a synergistic effect was observed in 12.00% of cases and an additive effect in 57.88% of cases. No pharmacological effect was observed in 30.13% of cases. At the high level of growth inhibition (MIC90 cut-off level), a synergistic effect was observed in 1.25% of cases, while an additive effect was observed in 23.81% of cases. No effect was observed in 74.94% of cases. It is worth noting that no presence of an antagonistic effect was observed in any of the trials at three different cut-off levels.

Analyzing clinical isolates and reference strains of *S. aureus*, Wang et al. [34] investigated the impact of cinnamic acid on the action of β-lactam antibiotics. They observed a synergistic effect of combining cinnamic acid with ampicillin, amoxicillin, oxacillin, and cefoxitin. In their studies, no additive or antagonistic effect was observed.

The potential synergistic effect was also investigated by Belmehdi et al. [35]. They analyzed a series of alcohol extracts of propolis—a product rich in carboxylic acids (including p-coumaric acid and ferulic acid)—and other derivatives. Analyzing the combination of ampicillin and propolis extracts against strains of *S. aureus* from the ATCC collection, they observed a synergistic effect three times. An additive effect was observed once. During this team’s research, determinations were also conducted for reference strains of *S. epidermidis* ATCC 12228, but no markings were made for the combination of extracts with β-lactams.

Ibrahim et al. conducted a similar analysis [36]. The effect of anise extract obtained from production residues was analyzed. Analyzing the activity of the combination of the extract with amoxicillin, they did not observe a synergistic effect. However, they observed an additive and antagonistic effect.

The evaluation of the synergistic effect was also conducted by Hemaiswarya et al. [37]. The activity of cinnamic acid, p-coumaric acid, and ferulic acid against strains of *S. aureus* was assessed. They observed a synergistic effect only in the combination of ampicillin with cinnamic acid. The combination of ampicillin with coumaric acid and ferulic acid showed an additive effect.

Derivatives of cinnamic acid exhibit a synergistic effect when combined with antibiotics, as confirmed by numerous studies demonstrating a positive impact (synergism) between cinnamaldehyde and the action of penicillin and ampicillin [38]. It is also worth mentioning that the synergistic effect of cinnamic acid derivatives extends beyond staphylococci. Hałasa et al. [39] verified the impact of cinnamic acid derivatives and ampicillin on strains of vancomycin-resistant *Enterococcus faecium*. They observed a synergistic effect for all derivatives.

The above studies seem to confirm the enhancement of β-lactam antibiotic activity in combination with cinnamic acid and its derivatives. The positive impact of the combination of carboxylic acids and β-lactam antibiotics not only appears to enhance the limited activity of β-lactams against hospital-acquired microorganisms but also justifies research into combinations of existing antibiotics with substances with limited therapeutic potential and provides hope for setting new standards in infection prevention and therapy. This seems particularly important in the face of increasing antibiotic resistance and depletion of antibiotic resources.

## 3. Experimental Section

### 3.1. Bacterial Strains

For the study, fifty clinical strains of *Staphylococcus epidermidis* were selected, which were isolated from vascular catheter infections in patients hospitalized in the Silesian Voivodeship (Poland). The species identification of all strains was performed using a mass spectrometer from bioMerieux (Vitec MS Prime^®^ system, Craponne, France). The assay was conducted in an external laboratory according to the manufacturer’s instructions. The procedure involved placing a smeared bacterial mass, directly obtained from a 24 h microbial culture, onto a dedicated plate. Subsequently, 1 µL of CHCA (alpha-cyano-3-hydroxycinnamic acid) solution was added. The resulting reaction mixture was allowed to dry. The assay was then programmed and placed in the VITEK^®^ MS PRIME device (bioMerieux, Craponne, France). Upon completion of the spectral analysis, all microorganisms were classified as *Staphylococcus epidermidis*. The *S. epidermidis* ATCC 12228 was used as the reference strain. Before starting the experiment, the strains were passaged twice to obtain a pure and fresh culture.

### 3.2. Determination of Antibiotic MIC Values

**Figure 8:**
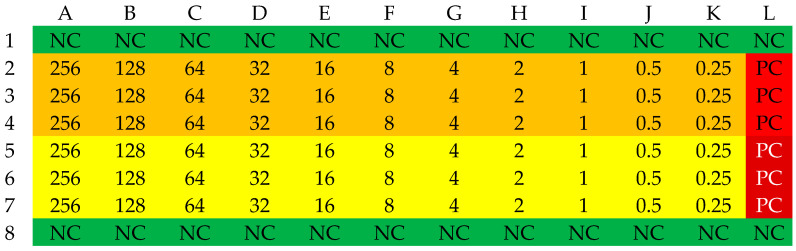
The organization scheme of a plate for determining the MIC values of an antibiotic. NC—Sterility control (green); PC—Growth control (red); yellow—first strain, orange—second strain. The numbers in the table correspond to the concentration of the antibiotic in the respective well [mg/L].

Selected β-lactam antibiotics in the powder for injection form were used to determine MIC values: ampicillin (Polfa Tarchomin, Warsaw, Poland), ampicillin/sulbactam (Swiss Parenterals LTD, Ahmedabad, India), cefazolin (Polpharma, Starogard Gdański, Poland), and cloxacillin (Polfa Tarchomin, Warsaw, Poland). The determination of MIC values was conducted in 96-well microtiter plates. The plate was divided into four zones: a sterility control zone, a growth control zone, and two test zones where the antibiotic was present at concentrations ranging from 256 to 0.25 mg/L. Sensitivity determination was performed three times for each strain. A series of dilutions of each antibiotic was prepared in 100 µL of Mueller–Hinton broth. Column H served as a growth control. Rows labeled 1 and 8 served as sterility controls. The areas in rows 2–4 and 5–7 were designated for the experiment. The layout of the microtiter plate is shown in Figure 8. In wells 2A–4L and 5A–7L, 100 µL of suspensions of the analyzed strains at a concentration of 1–2 × 10^8^ CFU/mL were placed, corresponding to a turbidity of 0.5 on the McFarland scale. A densitometer (Densilameter II from Erba Lachema, Brno, Česká republika) was used to determine the density. The suspension was prepared from colonies in the logarithmic phase, obtained from a 24 h bacterial culture on Columbia Blood Agar at a temperature of 37 °C.

The plates were incubated at 37 (±1) °C for 24 (±2) hours. Readings were conducted using a Multiskan EX Microplate Reader (Thermo Electron Corp., Vantaa, Finland) at a wavelength of 600 nm. The MIC value was determined as a 90% decrease in absorbance compared to the growth control [40].

### 3.3. Determination of MIC Values of Selected Carboxylic Acids 

**Figure 9:**
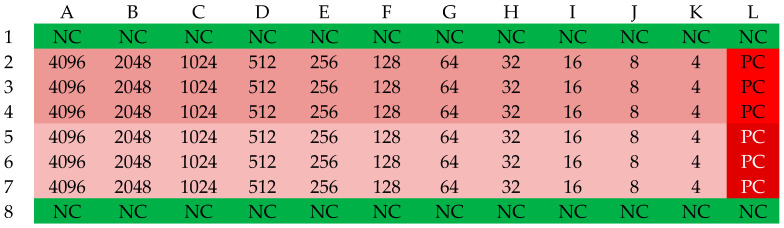
The organization scheme of a plate for determining the MIC values of an acid. NC—sterility control (green); PC—growth control (red); pink—first strain; deep pink—second strain. The numbers in the table correspond to the concentration of the antibiotic in the respective well [mg/L].

The determination of the MIC values of cinnamic acid (Aldrich Chemistry, Saint Louis, MO, USA), ferulic acid (Aldrich Chemistry, Saint Louis, MO, USA), sinapic acid (Aldrich Chemistry, Saint Louis, MO, USA), and p-coumaric acid (Aldrich Chemistry, Saint Louis, MO, USA) was conducted analogously to the determination of the MIC values of the selected antibiotics. To 100 µL of Mueller–Hinton broth, 100 µL of the carboxylic acid solution in Mueller–Hinton broth and ethanol were added. The ratio of ethanol to Mueller–Hinton broth was 1:4 and was determined experimentally. This ratio allowed for the creation of a clear suspension with a concentration of 8.192 mg/L. The prepared solution was used to develop a series of dilutions, which allowed for the creation of a substrate containing the tested acid at concentrations ranging from 4096 to 4 mg/L. Subsequently, the substrates (wells 2A-4L and 5A-7L) were inoculated with 100 µL of suspension of the tested strains with a density of 0.5 on the McFarland scale, prepared in the same way as in the previous stage of work. The MIC value determination was performed three times for each strain. Growth control and sterility control were organized in the same way as in the determination of antibiotic MIC values. Cultures were incubated at 37 (±1) °C for 24 (±2) hours. Readings were conducted using a Multiskan EX Microplate Reader (Thermo Electron Corp., Vantaa, Finland) at a wavelength of 600 nm. The MIC value was determined as a 90% decrease in absorbance compared to the growth control. The schematic organization of the 96-well plate is presented in Figure 9 [40,41].

### 3.4. Determination of FIC Index

**Figure 10:**
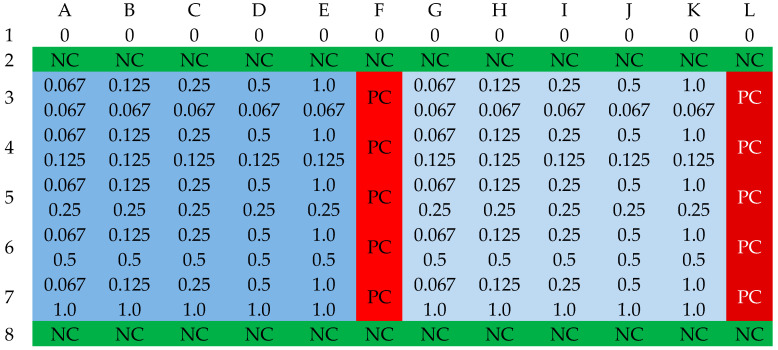
The organization scheme of a plate for determining the FIC values for an antibiotic and acid. NC—sterility control (green); PC—growth control (red); blue—first strain; deep blue—second strain. Cell notation scheme: (MIC of an antibiotic)/(MIC of an acid) [mg/L].

Determining the FIC index value required establishing the MIC values of both the β-lactam antibiotic and cinnamic acid or its derivatives and comparing them to assess their ability to inhibit microorganisms. The microtiter plate was divided into two sectors—one for each strain. Rows 1 and 8 served as sterility controls, and the growth control was determined in columns F and L. The MIC values of both compounds ranged from 1/16 to 1 MIC and were arranged according to the scheme presented in Figure 10.

In each well, 90 µL of the β-lactam antibiotic solution and 90 µL of the test acid solution were added. Carboxylic acids were dissolved in a mixture of Mueller–Hinton broth and ethanol. The ratio of ethanol to broth was 1:4. Subsequently, 10 µL of bacterial suspension with a turbidity of 0.5 on the McFarland scale was introduced. Cultures were incubated at 37 (±1) °C for 24 (±2) hours. Readings were conducted using a Multiskan EX Microplate Reader (Thermo Electron Corp., Vantaa, Finland) at a wavelength of 600 nm. The MIC value was determined as a 90%, 75%, and 50% decrease in absorbance compared to the growth control [42].

### 3.5. Interpretation of FIC Determination Results 

The obtained absorbance values were subtracted from the background value (absorbance of the sterility control). The numerical value was referenced to the growth control value. No results exceeding 10% of the growth control value were considered in further analysis. The next step involved determining the MIC value of the antibiotic in the presence of the acid and the MIC value of the acid in the presence of the antibiotic. The obtained values were referenced to the equation presented below.

(1) $${\mathrm{FIC}}_{\mathrm{index}}=\frac{MIC\;of\;antibiotic\;in\;combintaion}{MIC\;of\;antibiotic\;alone}+\frac{MIC\;of\;acid\;in\;combination}{MIC\;of\;acid\;alone}$$

The result of this equation was classified into one of four categories of pharmacological effects:

- Synergy (FIC index ≤ 0.5);
- Additive effect (0.5 < FIC index ≤ 1);
- Lack of pharmacological effect (1 < FIC index ≤ 4);
- Antagonistic effect (FIC index > 4) [43].

## 4. Conclusions

Infections caused by *Staphylococcus* spp. present a challenge due to high resistance to β-lactam antibiotics. Cinnamic acid and its derivatives, including sinapic acid, ferulic acid, and p-coumaric acid, show limited antibacterial activity individually—MIC values at the level of 2048 and 4096 mg/L. However, when combined with β-lactam antibiotics, they often exhibit enhanced efficacy, often through synergistic effects. The combination of all tested substances—cinnamic acid and its derivatives—with ampicillin resulted in a 50% reduction in growth in more than half of the tested strains. In turn, the combination of these substances with cloxacillin resulted in a 50% reduction in growth in over 46% of the strains tested. These findings emphasize the significance of investigating novel combinations of β-lactams and cinnamic acid derivatives to develop innovative therapeutic strategies.

## Data Availability

Data are contained within the article and Supplementary Materials.

## Supplementary Materials

The following supporting information can be downloaded at: https://www.mdpi.com/article/10.3390/antibiotics13080710/s1, Table S1: Percentage values of FICi for different antibiotics and acids; Table S2: MIC values of cinnamic acid and it’s derivatives among strains; Table S3: MIC values of antibiotics among strains.
