# Supplementary material for: Synergistic Interactions between Selected β-Lactam Antibiotics and Cinnamic Acid and Its Chosen Derivatives
Source: Antibiotics (Basel). 2024 Jul 29;13(8):710. doi: 10.3390/antibiotics13080710 (PMC11350685; doi:10.3390/antibiotics13080710)
Supplement: Supplementary file 1 [file antibiotics-13-00710-s001.zip › Table S1.pdf]

Table S1. Percentage values of FICi for different antibiotics and acids; 1 - ampicillin; 2 - ampicillin/sulbactam; 3 - cefazolin; 4 - cloxacillin; S - synergistic effect; A - additive effect; N - no pharmacological effect.

		**cinnamic acid**			**p-coumaric acid**			**ferulic acid**			**sinapic acid**		
		**MIC 50%**	**MIC 75%**	**MIC 90%**	**MIC 50%**	**MIC 75%**	**MIC 90%**	**MIC 50%**	**MIC 75%**	**MIC 90%**	**MIC 50%**	**MIC 75%**	**MIC 90%**
**1**	**S**	58	4	0	78	32	4	70	16	2	68	12	0
	**A**	12	34	18	18	52	24	20	66	24	28	52	16
	**N**	30	62	82	4	16	72	10	18	74	4	36	84
**2**	**S**	26	4	0	26	2	0	8	2	0	40	8	0
	**A**	52	52	25	56	60	16	52	40	30	56	82	42
	**N**	22	44	75	18	38	84	40	58	70	4	10	58
**3**	**S**	54	10	0	44	6	4	20	18	2	50	22	0
	**A**	44	68	16	52	68	34	56	26	34	46	66	22
	**N**	2	22	84	4	26	62	24	56	64	4	12	78
**4**	**S**	58	8	6	50	10	0	46	10	2	56	28	0
	**A**	36	44	14	42	78	32	44	66	12	44	72	22
	**N**	6	48	80	8	12	68	10	24	86	0	0	78
