# Supplementary material for: Synergistic Interactions between Selected β-Lactam Antibiotics and Cinnamic Acid and Its Chosen Derivatives
Source: Antibiotics (Basel). 2024 Jul 29;13(8):710. doi: 10.3390/antibiotics13080710 (PMC11350685; doi:10.3390/antibiotics13080710)
Supplement: Supplementary file 1 [file antibiotics-13-00710-s001.zip › Table S2.pdf]

Table S2. MIC values of cinnamic acid and it's derivatives among strains.

strain	cinnamic acid [μg/mL]	ferulic acid [μg/mL]	p-coumaric acid [μg/mL]	sinapic acid [μg/mL]	strain	cinnamic acid [μg/mL]	ferulic acid [μg/mL]	p-coumaric acid [μg/mL]	sinapic acid [μg/mL]
**1**	4096	4096	4096	4096	**26**	4096	4096	4096	4096
**2**	4096	4096	4096	4096	**27**	2048	4096	2048	4096
**3**	2048	4096	4096	4096	**28**	2048	4096	4096	4096
**4**	2048	4096	4096	4096	**29**	4096	4096	4096	4096
**5**	4096	4096	4096	4096	**30**	4096	4096	4096	4096
**6**	4096	4096	4096	4096	**31**	4096	4096	4096	4096
**7**	4096	4096	4096	4096	**32**	4096	4096	4096	4096
**8**	4096	4096	4096	4096	**33**	4096	4096	4096	4096
**9**	4096	4096	2048	4096	**34**	2048	4096	4096	4096
**10**	4096	4096	4096	4096	**35**	4096	4096	2048	4096
**11**	4096	4096	4096	4096	**36**	4096	4096	4096	4096
**12**	4096	4096	4096	4096	**37**	4096	4096	4096	4096
**13**	4096	4096	4096	4096	**38**	4096	4096	4096	4096
**14**	4096	4096	4096	4096	**39**	4096	4096	4096	4096
**15**	4096	4096	4096	4096	**40**	4096	4096	4096	4096
**16**	4096	4096	4096	4096	**41**	4096	4096	4096	4096
**17**	4096	4096	4096	4096	**42**	2048	4096	4096	4096
**18**	4096	4096	4096	4096	**43**	4096	4096	4096	4096
**19**	4096	4096	4096	4096	**44**	4096	4096	4096	4096
**20**	4096	4096	4096	4096	**45**	4096	4096	4096	4096
**21**	4096	4096	4096	4096	**46**	4096	4096	4096	4096
**22**	4096	4096	4096	4096	**47**	4096	4096	4096	4096
**23**	4096	4096	4096	4096	**48**	4096	4096	4096	4096
**24**	4096	4096	4096	4096	**49**	2048	4096	4096	4096
**25**	4096	4096	4096	4096	**50**	2048	4096	4096	4096
