# Supplementary material for: Synergistic Interactions between Selected β-Lactam Antibiotics and Cinnamic Acid and Its Chosen Derivatives
Source: Antibiotics (Basel). 2024 Jul 29;13(8):710. doi: 10.3390/antibiotics13080710 (PMC11350685; doi:10.3390/antibiotics13080710)
Supplement: Supplementary file 1 [file antibiotics-13-00710-s001.zip › Table S3.pdf]

Table S3. MIC values of antibiotics among strains.

strain	ampicillin [μg/mL]	ampicillin-sulbactam [μg/mL]	cefazolin [μg/mL]	cloxacillin [μg/mL]	strain	ampicillin [μg/mL]	ampicillin-sulbactam [μg/mL]	cefazolin [μg/mL]	cloxacillin [μg/mL]
**1**	256	32	64	512	**26**	0.25	0.25	0.5	0.25
**2**	256	32	256	512	**27**	256	64	256	512
**3**	128	32	128	512	**28**	512	256	0.5	0.25
**4**	512	32	16	64	**29**	128	64	64	128
**5**	512	32	128	512	**30**	256	128	16	16
**6**	512	32	256	512	**31**	512	8	1	0.5
**7**	256	32	64	512	**32**	256	64	128	512
**8**	256	32	1	256	**33**	0.25	0.25	0.25	0.25
**9**	1	8	0.25	0.25	**34**	512	32	64	128
**10**	512	4	2	0.5	**35**	256	8	0.5	0.5
**11**	512	16	32	512	**36**	0.25	0.25	0.25	0.25
**12**	256	64	256	512	**37**	64	16	16	512
**13**	16	1	0.25	0.25	**38**	256	32	128	512
**14**	256	32	64	128	**39**	256	32	128	512
**15**	256	32	64	128	**40**	128	4	0.5	0.5
**16**	256	32	16	64	**41**	256	32	128	512
**17**	512	256	64	256	**42**	256	8	1	0.5
**18**	256	16	0.5	0.25	**43**	256	32	2	0.5
**19**	256	32	16	256	**44**	256	64	256	256
**20**	512	64	0.5	0.5	**45**	256	64	256	512
**21**	256	64	64	256	**46**	512	32	128	512
**22**	256	16	0.5	0.25	**47**	32	32	64	512
**23**	256	64	256	512	**48**	512	64	256	512
**24**	256	32	1	0.25	**49**	512	128	256	512
**25**	256	32	64	512	**50**	512	64	128	128
